# Effects of Dietary Supplementation of DL-Methionine or DL-Methionine Hydroxyl Analogue (MHA-Ca) on Growth Performance and Blood and Liver Redox Status in Growing Pigs

**DOI:** 10.3390/ani14233397

**Published:** 2024-11-25

**Authors:** Emmanuel O. Oladejo, Md Shamimul Hasan, Susan C. Sotak, John K. Htoo, James Brett, Jean M. Feugang, Shengfa F. Liao

**Affiliations:** 1 Department of Animal and Dairy Sciences, Mississippi State University, Starkville, MS 39759, USA; 2 Evonik Operations GmbH, Rodenbacher Chaussee 4, 63457 Hanau-Wolfgang, Germany; 3 College of Veterinary Medicine, Mississippi State University, Starkville, MS 39759, USA

**Keywords:** methionine, methionine hydroxyl analogue, growth performance, oxidative stress, antioxidative status, pig

## Abstract

Oxidative stress impairs pigs’ health and production performance. This study examined the effects of level and source of methionine (Met) supplementation against the diquat-induced oxidative stress in pigs. Forty growing barrows were randomly assigned to four groups. Groups 1 and 2 received a basal diet that was adequate in energy and nutrients (Diet 1), Group 3 received a DL-Met-supplemented diet, and Group 4 received an MHA-Ca-supplemented diet. After 3 weeks of feeding (Phase 1), pigs were injected with 10 mL saline (Group 1) or 10 mg/kg-BW diquat in 10 mL saline (Groups 2, 3, and 4), and then continually fed for one week (Phase 2). During or at the end of Phase 1, there were no differences in nearly all of the growth performance and redox parameters among the four groups. During or at the end of Phase 2, however, the ADFI and ADG were lower (*p* < 0.01) in Group 2 than in Group 1 while the serum malondialdehyde content was higher (*p* < 0.05) in Group 2 than in Group 1. The serum glutathione peroxidase (GPX) activity and liver superoxide dismutase (SOD) activity were lower (*p* < 0.05) in Group 2 than in Group 1. The serum GPX activity, the serum total antioxidant capacity, and the liver GPX activity were higher (*p* < 0.05) in Group 3 than in Group 2. There were nearly no differences in these redox parameters between Groups 3 and 4. In conclusion, MHA-Ca and DL-Met, when used at an equivalent bioefficacy level, had similar effects on the pigs’ growth performance and antioxidative status. Therefore, increased Met supplementation can improve pigs’ antioxidative status.

## Simple Summary

This study aimed to compare the protective roles of two commercial methionine (Met) products, DL-Met and MHA-Ca, supplemented at an equal bioefficacy level in the diets of growing pigs. The results showed that the diquat injection in pigs induced oxidative stress by reducing growth performance and antioxidative status. Although supplementing with 25% more DL-Met than the NRC (2012) recommendation did not improve growth performance, it enhanced the pigs’ antioxidative status. Additionally, this study indicated no differences in the influence of MHA-Ca vs. DL-Met on the growth performance and antioxidative status in these pigs. These findings suggest that both Met products similarly affected the antioxidative status of the pigs.

## 1. Introduction

As a sulfur-containing amino acid (SAA), methionine (Met) exerts numerous biochemical and physiological functions to regulate pig growth, development, reproductive performance, and health, in addition to functioning as a building block for protein biosynthesis [1,2]. For example, Met serves as a methyl donor for DNA methylation, and is a precursor for the synthesis of several bioactive compounds, such as cysteine (Cys), which in turn is required for the synthesis of glutathione (GSH), a major intracellular antioxidant, and a precursor AA of taurine [2]. Crystalline Met, such as anhydrous DL-Met, is commonly supplemented to swine diets to meet the swine nutritional requirements for SAA (i.e., Met + Cys). Commercial feed-grade Met sources also include L-Met and DL-Met hydroxyl analogue (DL-MHA; D,L-2-hydroxy-4-methylthiobutanoic acids). DL-MHA is chemically different from DL-Met as the characteristic nitrogen-containing amino group is replaced by a hydroxy group [2]. The liquid free acid form of DL-MHA (MHA-FA) usually contains 12% water and 88% MHA-FA molecules, of which about 23% is present in the dimer and oligomer forms. Commercial MHA products also include MHA-Ca, a solid calcium salt powder, synthesized by neutralizing MHA-FA with calcium hydroxide or calcium oxide. MHA-Ca usually contains 12 to 14% calcium, and is more stable for industry application [2,3].

Numerous studies in chickens have shown that the dietary supplementation of Met can increase the chickens’ antioxidant capacity [4,5], and that different forms of Met products can affect the antioxidant capacity differently [6]. However, reports concerning the effects of crystalline DL-Met or MHA-Ca on the antioxidative status of pigs are scarce. Given the fact that the oxidation of lipids, proteins, and DNA in the body can negatively affect pig health and growth performance [7], there is a critical need to determine the impacts of the dietary supplementation of DL-Met or MHA on pig health and growth performance as well as the associated underlying mechanisms.

Diquat (dibromide monohydrate) is a bipyridyl herbicide that can be intraperitoneally injected to pigs to induce oxidative stress (OS), which exerts inhibitory effects on pig nutrient utilization and growth performance [8,9]. Thus, the overall goal of this study was to investigate the protective roles of dietary MHA-Ca vs. DL-Met on the diquat challenged pigs. The two specific objectives of this study were to test the effectiveness of the dietary supplementation of MHA-Ca vs. DL-Met (at 25% above the requirement) on (1) the growth performance, and (2) the antioxidative status of the young growing pigs. The hypotheses for these objectives state that (1) the dietary supplementation of Met or MHA-Ca above the NRC [10] recommended requirement would enhance the pigs’ antioxidative status and growth performance, and (2), the effects of these two Met products based on an average bioefficacy of 65% for MHA-Ca relative to DL-Met [3,11] on the pigs’ antioxidative status and growth performance would be the same.

## 2. Materials and Methods

### 2.1. Animal Trials and Growth Performance Measurements

Forty crossbred (Large White × Landrace) young barrows (around 7 weeks of age) purchased from a local commercial farm were transferred to an environment-controlled swine barn at the Leveck Animal Research Center of Mississippi Agricultural and Forestry Experiment Station and used in two feeding trials (as two experimental blocks) according to a randomized complete block design with the pig or pen as the experimental unit. Upon arrival, pigs were fed a commercial nursery diet until their body weights (BWs) reached 25 kg (on average), during which period the pigs were allowed ad libitum access to the diet and fresh water. Within each block, the barrows were randomly assigned to 20 individual pens, and further randomly assigned to four treatment groups (n = 5) fed three different experimental diets (Table 1). The calculated energy and nutrient composition of the three experimental diets are shown in Table 2, while the laboratory-analyzed energy and nutrient composition are shown in Supplementary Table S1.

Groups 1 and 2 received a basal diet (Diet 1) that was formulated based on corn and soybean meal to meet or slightly exceed the recommended requirements of energy and nutrients for 25 to 50 kg pigs according to the NRC [10] and AMINODat 6.0 [12]. Group 3 received a DL-Met supplemented diet (Diet 2) in which the concentration of standard ileal digestible (SID) Met + Cys was 125% of that in Diet 1. Group 4 received an MHA-Ca supplemented diet (Diet 3) in which the amount of MHA-Ca added was 1.54 × the amount (in weight) of the DL-Met added in Diet 2 (based on the average bioefficacy of MHA-Ca being 65% relative to DL-Met [13]). All three diets (in mash form) contained the same amount of net energy (NE) that met or slightly exceeded 2475 kcal/kg, the NRC [10] recommended level. Additionally, the SID lysine was adequate at 1.00% in all three diets [13]. The NE, SID CP, and SID AA values of the ingredients calculated based on the laboratory analyses were used for the diet formulations. Drinking water was freely available for all pigs throughout the feeding trials.

After 3 weeks (on day 22) of experimental feeding (Phase 1), the pigs were injected intraperitoneally with either 10 mL sterile saline (for Group 1) or 10 mg/kg-BW diquat in 10 mL saline (for Groups 2, 3, and 4). According to previous publications, diquat injection can induce OS in pigs [8,9]. After the diquat injection, pigs were continually fed with their respective diets for 7 more days (Phase 2) before being sent to a slaughter facility (i.e., the Mississippi State University Meat Science and Muscle Biology Laboratory) for tissue sample collection.

During the entire animal trial period, the feed intake was recorded, and all pigs were weighed on day 1 (the start of Phase 1), day 22 (the end of Phase 1 or the start of Phase 2), and on day 29 (the end of Phase 2). The daily gain in BW, average daily gain (ADG), average feed intake (ADFI), and gain-to-feed (G:F) ratio were calculated accordingly. All experimental protocols involving the caring, handling, and treatment of pigs were subjected to approval by the Mississippi State University Institutional Animal Care and Use Committee (approval number: IACUC-19-021).

### 2.2. Tissue Sample Collection and Laboratory Analysis

Tissue samples were collected from all animals on day 1, immediately before being fed the experimental diets; on day 22, a day before the saline and diquat injections; and on day 29, the end of the animal trial. In each of two animal trials, blood samples were collected, in the mornings (between 0700 and 0900 h), from all pigs via jugular venipuncture, and kept on ice for approximately 30 to 60 min. All samples displayed clot formation were centrifuged (2000× *g*, 4 °C, 10 min) for serum collection. The serum samples were then stored at −80 °C until laboratory analyses. On day 29, the pigs were euthanized (after 12-h fasting) at the Mississippi State University Meat Science and Muscle Biology Laboratory by using a captive bolt following IM injection of stresnil and xylazine (2 and 4 mg/kg BW, respectively). After euthanasia, the abdomen was opened, and the liver was removed from the carcass. The liver samples were taken from the right medial lobe (3 to 5 g/each), snap-frozen in liquid nitrogen, and stored at −80 °C until laboratory redox status analyses [14].

Both the serum and liver samples were used to measure the activities of superoxide dismutase (SOD), catalase (CAT), and glutathione peroxidase (GPX), the total antioxidant capacity (TAC) as well as the contents of malondialdehyde (MDA) and glutathione (GSH) using the respective commercial colorimetric assay kit as instructed by the manufacturers (Cayman Chemical Company; Ann Arbor, MI, USA).

### 2.3. Data Statistical Analysis

Data were statistically analyzed using the general linear model procedure (PROC GLM) of SAS (version 9.4; SAS Institute Inc., Cary, NC, USA) for the analysis of variance (ANOVA) with the block (i.e., trial) and treatment as the main effects. On all the growth performance parameters, there were no differences between the two blocks. Thus, the block was not included as a variable in all the ANOVA analyses. The means of the treatment effects were separated by three pre-planned orthogonal contrast tests: Group 2 vs. Group 1, Group 3 vs. Group 2, and Group 4 vs. Group 3. *p* ≤ 0.05 was considered significant, and 0.05 < *p* ≤ 0.10 as a tendency.

## 3. Results and Discussion

### 3.1. Growth Performance of the Pigs

Table 3 shows that during Phase 1 (before the diquat injection), there were no differences (*p* > 0.10) in the initial BW, final BW, ADFI, and ADG among the four dietary treatment groups, which indicates that 25% more Met supplementation (either in the form of DL-Met or MHA-Ca) did not further improve the growth performance of the young growing pigs. However, in terms of the G:F ratio, although there was no difference between Group 1 through to Group 3, the ratio in Group 4 was lower (*p* < 0.05) than that in Group 3. This result indicates that the MHA-Ca used in this study was not as efficient as DL-Met (based on an average bioefficacy of 65% for MHA-Ca relative to DL-Met) in supporting the pigs to convert feed to body mass.

During Phase 2 (after the diquat injection), the AFDI (−30%) and ADG (−46%) in Group 2 were significantly lower (*p* < 0.01) than those in Group 1, and the final BW in Group 2 tended to be lower (*p* < 0.10) than that in Group 1 (Table 3). Although not significant (*p* = 0.13), the G:F ratio was also reduced (−28%) by the diquat challenge. These results indicate that the pigs in Group 2 were clearly under OS caused by diquat injection. Table 3 also shows that there were no differences in the ADFI, ADG, G:F ratio, and final BW between Groups 3 vs. 2 or between Groups 4 vs. 3 during Phase 2, which suggests that more Met supplementation (either in the form of DL-Met or MHA-Ca) did not bring back the compromised growth performance of the pigs due to the OS induced by diquat injection (as shown in Group 2).

### 3.2. Antioxidative Status of the Pigs

#### 3.2.1. Malondialdehyde (MDA) Content in Serum and Liver

The data on days 1 and 22 (before the diquat injection) show that there were no differences (*p* > 0.10) in the serum MDA contents among the four treatment groups, and the data on day 29 (after the diquat injection) showed no differences (*p* > 0.10) in the liver MDA contents among the four treatment groups (Table 4). However, the serum MDA content in Group 2 was higher (*p* = 0.01) than that in Group 1 on day 29, which indicates that the pigs in Group 2 were oxidatively stressed after diquat injection. The data (Table 4) further showed that more Met supplementation in either the DL-Met form (in Group 3) or MHA-Ca form (in Group 4) did not reduce (*p* > 0.10) the MDA contents in the serum or liver.

Serum MDA content is commonly used as a biomarker for the OS-resulted damage such as lipid peroxidation in the body, and the increased serum MDA content in Group 2 of this study indicates some OS-resulted damage induced by diquat injection to these pigs. Supplementation of 25% more Met for the pigs in Groups 3 and 4 (in the DL-Met and MHA-Ca forms, respectively) did not attenuate the OS-resulted lipid peroxidation. This might be due to the limited capacity of 25% more Met to protect lipids from oxidative damage in the body.

#### 3.2.2. Total Antioxidant Capacity (TAC) in Serum

Table 5 shows that on days 1 and 22 (before the diquat injection), there were no differences (*p* > 0.10) in the TAC measurements among the four treatment groups. On day 29 (after the diquat injection), however, the TAC of Group 3 was increased (*p* = 0.05) when compared to Group 2, which indicates that more DL-Met supplementation improved the serum TAC activity in the oxidatively stressed pigs. There was no difference (*p* > 0.10) in the TAC measurements between Group 4 and 3 on day 29, which indicates that the MHA-Ca (at 65% bioefficacy relative to DL-Met) and DL-Met were equally effective in the aspect of enhancing the serum TAC activity in the oxidatively stressed pigs.

TAC is an important biomarker that provides a sign about the antioxidant status in the animal body, although it does not account for the role of antioxidant enzymes such as SOD, GPX, and CAT [15,16]. The data of the serum TAC measurements in this study suggest that more Met supplementation (either in the form of DL-Met or MHA-Ca) can increase the TAC in oxidatively stressed pigs.

#### 3.2.3. Glutathione (GSH) Content in Serum

Table 6 shows that on day 1 and day 22, there were no differences (*p* > 0.10) in the serum GSH contents among the four dietary treatment groups, which indicates that more dietary Met supplementation (either in the form of DL-Met or MHA-Ca) did not alter the serum GSH content in normal (i.e., non-diquat challenged) growing pigs during the 3 weeks of feeding. The serum GSH contents on day 29 were not analyzed because a few serum samples had run out.

The serum level of GSH is an indicator of animal antioxidative capacity, and the data in Table 6 do not indicate whether more Met supplementation (either in the form of DL-Met or MHA-Ca) would improve the antioxidative capacity performed by the molecules of GSH in growing pigs.

#### 3.2.4. Glutathione Peroxidase (GPX) Activity in Serum and Liver

Table 7 shows that on day 1 and day 22 (before the diquat injection), there were no differences (*p* > 0.10) in the serum GPX activity between Groups 2 and 1. However, on day 29 (one week after the diquat injection), both the serum and liver GPX activities were reduced at a tendency level (*p* ≤ 0.10), which indicates that the GPX activity might be reduced in oxidatively stressed pigs.

Comparing Group 3 to Group 2 (Table 7), both the serum and liver GPX activities were significantly increased *(p* < 0.05), which indicates that more DL-Met supplementation can increase the GPX activity in the pigs oxidatively stressed by the diquat injection. When comparing Group 4 to Group 3, the serum GPX activity on day 29 tended to reduce (*p* < 0.10), which indicates that the MHA-Ca product used in this study may not be as efficient as DL-Met in terms of enhancing the compromised GPX activity in the oxidatively stressed pigs (Table 7).

GPX is a class of antioxidant enzymes that helps to maintain intracellular homeostasis by preventing lipids from peroxidation during an OS period. The likely declined GPX activity in the serum and liver in Group 2 pigs indicates some OS-resulted damage induced by diquat injection. Supplementation of 25% more Met (in the DL-Met form) enhanced the antioxidative capacity through GPX in the oxidatively stressed pigs (Group 3). However, the metabolism of MHA-Ca might not be as efficient as DL-Met (on an equal bio-efficacy level) in stimulating the GPX activity in the body.

#### 3.2.5. Superoxide Dismutase (SOD) Activity in Serum and Liver

Table 8 shows that on either day 1, day 22, or day 29 of the experiment, there were no differences (*p* > 0.10) in the serum SOD activity among the four dietary treatment groups, which suggests that the diquat injection did not reduce the serum SOD activity, and that either more DL-Met or more MHA-Ca did not enhance the serum SOD activity.

In terms of liver SOD activity, the activity in Group 2 was reduced *(p* < 0.05) when compared to Group 1, which indicates that the OS induced by the diquat injection reduced the liver SOD activity. The liver SOD activity in Group 3 tended to increase (*p* = 0.08) when compared to Group 2, which indicates that more dietary DL-Met likely increased the liver SOD activity in these oxidatively stressed pigs. There was no difference (*p* > 0.10) in the liver SOD activity between Group 4 and Group 3, which suggests that the MHA-Ca product used in this study was as efficient as DL-Met (on an equal bio-efficacy level) in enhancing the liver SOD activity in the oxidatively stressed pigs.

SOD, an antioxidant enzyme, is also a broadly used biomarker for the antioxidative capacity in the body, and the decreased liver SOD in Group 2 pigs indicates some OS-resulted damage induced by diquat injection. Supplementation of 25% more Met for the pigs in Groups 3 and 4 (in the DL-Met and MHA-Ca forms, respectively) might be efficient to attenuate the OS-resulted damages in the body via the SOD enzyme.

#### 3.2.6. Catalase (CAT) Activity in Serum and Liver

Table 9 shows that there were no differences (*p* > 0.10) in either the serum or the liver CAT capacity between Group 2 and Group 1 at any time, which indicates that the diquat injection did not affect the serum or liver CAT activity. Comparing Group 3 to Group 2, a reduced serum CAT activity (*p* < 0.05) was detected on day 22, and there was no difference (*p* = 0.53) in serum CAT activity on Day 29 between Groups 4 and 3. These two results indicate that feeding more DL-Met or MHA-Ca to the pigs for about 3 weeks reduced the serum CAT activity. However, more Met supplementation (either in the form of DL-Met or MHA-Ca) attenuated the reduced serum or liver CAT activity in the oxidatively stressed pigs.

CAT, an antioxidant enzyme, is also commonly used as a biomarker for the antioxidative capacity in pigs. Why more Met supplementation for 3 weeks might lead to a reduction in serum CAT activity requires further investigation.

### 3.3. Overall Discussion

Diquat, known for its herbicidal properties, has been used to induce OS status in animals [9,17]. This study clearly showed that the diquat injection must have induced OS in the growing pigs, which was evidenced by the increased serum MDA level, the reduced liver SOD activity as well as the possibly reduced serum and liver GPX activities (Table 2, Table 7 and Table 8). The growth performance of these pigs was also compromised as both the ADFI and ADG were reduced after the diquat injection (Table 3). Consistent with our findings in this study, some previous studies on pigs have also reported lower levels of GPX, CAT, and SOD, along with higher levels of MDA under the OS status in pigs [18,19,20] As is known, under an OS condition, the body’s antioxidative capacity must not be high enough to counteract the excessively produced reactive oxygen species (ROS), causing damage to crucial cell components [21] such as those in small intestinal epithelial cells [22]. In response to this cellular damage, pigs may try to lower their metabolic burden by reducing their feed intake [22], which can negatively affect their growth performance, as reported in several previous studies [9,17,23].

As comprehensively reviewed by us in 2020 [2], Met possesses numerous biochemical and physiological functions that include its direct and indirect antioxidative properties, which can scavenge almost all oxidizing molecules including H_2_O_2_, hydroxyl radicals, peroxynitrite, chloramines, and hypochlorous acid in the animal body [24,25,26]. One objective of this study was to examine whether supplementing a corn- and soybean meal-based diet (Table 2) with 25% more SID Met + Cys than the level recommended by the NRC [10] could enhance the antioxidative capacity and growth performance of the oxidatively stressed pigs. Our results showed that the growth performance of the oxidatively stressed pigs was not improved with the dietary supplementation of more SID Met + Cys (Table 3). However, this study found a possible increase in SOD levels in the liver (Table 8), along with a higher TAC in the serum (Table 5) and increased GPX activities in both the serum and liver (Table 7) in the oxidatively stressed pigs. To date, there has been limited research concerning the antioxidative effects of Met supplementation in pigs. Estévez et al. [5] reported that supplementing SAA above its nutritional requirement could protect muscle lipids and proteins from oxidation in pigs. Consistent with our findings, a 30% increase in L-Met in the diet for low-weight-birth piglets reduced the MDA levels and increased the GSH content in their muscles [27]. Additionally, the increased liver GSH content was observed in DL-Met-fed weaned pigs challenged with lipopolysaccharides [28].

As stated in the introduction section, MHA-Ca is an alternative, cost-effective source of Met for animal feed [13,29]. Another objective of this study was to compare the effects of the dietary supplementation of MHA-Ca vs. DL-Met (based on an average bioefficacy of 65% for MHA-Ca relative to DL-Met) on the growth performance and antioxidant status in oxidatively stressed pigs (induced by diquat injection). The results from Phase 2 of this study showed no differences between the dietary supplementations of MHA-Ca vs. DL-Met on growth performance and all of the antioxidative parameters tested in the oxidatively stressed pigs (Table 3, Table 4, Table 5, Table 6, Table 7, Table 8 and Table 9). This finding suggests that there were no differences between MHA-Ca and DL-Met in affecting the growth performance and antioxidative status in growing pigs when using an average bioefficacy of 65% for MHA-Ca relative to DL-Met. Currently, no research has been found in the literature that has addressed the effects of MHA or MHA-Ca on the antioxidant status of pigs, though some studies have shown a better antioxidative status in poultry with higher GSH and GPX activities when MHA-supplemented diets were fed [6,13,30].

## 4. Conclusions

Our data showed that the intraperitoneal injection of diquat induced oxidative stress resulted in a drastically reduced growth performance and antioxidative capacity in the blood and liver of young growing pigs. Although dietary supplementation with 25% more DL-Met than the NRC [10] recommended level did not improve growth performance, the supplementation could enhance the antioxidative status of the pigs, as evidenced by the changed antioxidative biomarker levels in these oxidatively stressed pigs. Additionally, our findings also suggest that MHA-Ca and DL-Met, when supplemented at the same level, using an average bioefficacy of 65% for MHA-Ca relative to DL-Met, similarly affected the antioxidative status of the pigs, as evidenced by the levels of the antioxidative biomarkers showed in these oxidatively stressed pigs.

## Figures and Tables

**Table 1:** The composition of the three experimental diets used in this study ^1^.

	Composition (%)		
Ingredients	Diet 1	Diet 2	Diet 3
Corn	73.965	73.805	73.638
Soybean meal	22.770	22.770	22.770
L-Lysine-HCl, 78.8%	0.360	0.360	0.360
DL-Methionine	0.150	0.310	
MHA-Ca ^2^	–	–	0.477
L-Threonine	0.130	0.130	0.130
L-Tryptophan	0.040	0.040	0.040
Limestone	0.850	0.850	0.850
Dicalcium phosphate	1.400	1.400	1.400
Salt	0.200	0.200	0.200
Mineral premix ^3^	0.070	0.070	0.070
Vitamin premix ^4^	0.065	0.065	0.065
Diet, total	100.000	100.000	100.000

^1^ Diet 1 was the basal diet that met or slightly exceeded the NRC [10] recommended nutrient requirements. Diet 2 was formulated by adding more DL-methionine at the rate of 0.160% to the basal diet. Diet 3 was formulated by adding MHA-Ca at the rate of 0.477% to the basal diet to contain the same Met activity as the supplemented DL-Met content in Diet 2 (based on its bioefficacy of 65% in comparison with DL-Met). ^2^ MHA-Ca = a solid calcium salt of methionine hydroxyl analogue (MHA, 84%). The liquid free acid form of DL-MHA (MHA-FA) usually contains 88% of a mixture of mono-, di-, and oligomers of D,L-2-hydroxy-4-methylthiobutanoic acids. ^3^ Swine trace mineral (NB-8534; Nutra Blend, LLC, Neosho, MO, USA) premix was used. See Table 2 for the dietary mineral composition. ^4^ Swine vitamin premix (NP26873; DSM Nutritional Products Canada, Inc., Ontario, ON, Canada) was used. See Table 2 for the dietary vitamin composition.

**Table 2:** The calculated nutrient and energy composition of the three experimental diets^1^ on an as-fed basis.

	Requirement ^2^	Diet 1	Diets 2 and 3
Net energy (kcal/kg)	2440	2531	2534
Crude protein (%, SID)	14.49	14.49	14.57
Lys (%, SID)	1.00	1.00	1.00
Met + Cys (%, SID)	0.62	0.62	0.78
Thr (%, SID)	0.65	0.65	0.65
Trp (%, SID)	0.20	0.20	0.20
Arg (%, SID)	0.40	0.99	0.99
His (%, SID)	0.32	0.39	0.39
Leu (%, SID)	1.00	1.32	1.32
Ile (%, SID)	0.55	0.60	0.60
Val (%, SID)	0.68	0.68	0.68
Phe (%, SID)	0.60	0.73	0.73
Phe + Tyr (%, SID)	0.95	1.18	1.17
tCa (%)	0.66	0.67	0.67
tP (%)	0.56	0.56	0.56

^1^ The calculated mineral and vitamin contents (per kg of diet) were: Na, 1.06 g; Cl, 1.59 g; K, 6.58 g; Mg, 1.45 g; S, 1.59 g; Cu, 16.3 mg; Fe, 151 mg; I, 0.14 mg; Mn, 30.6 mg; Zn, 102 mg, Se, 0.22 mg; carotene, 1.48 mg; vitamin A, 2861 IU; vitamin D3, 358 IU; vitamin E, 28.5 IU; vitamin K, 1.14 mg; vitamin B1, 2 31 mg; vitamin B2, 3.55 mg; niacin, 35.6 mg; vitamin B5, 13.0 mg; vitamin B6, 5.16 mg; biotin, 0.10 mg; folacin, 0.42 mg; vitamin B12, 10.0 µg, and choline, 1.46 mg. ^2^ The energy and nutrient requirements were adopted from the NRC [10] and the essential amino acid ratios were adjusted based on the recommendation by AMINODat 6.0 [12].

**Table 3:** Effects of dietary supplementation of DL-Met or MHA-Ca on the growth performance of the growing pigs ^1^.

		Diquat Challenged ^2^				Contrast *p*-Value ^3^		
Item	G 1	G 2	G 3	G 4	SEM	G 2 vs. G 1	G 3 vs. G 2	G 4 vs. G 3
*Phase 1 (Days 1 to 22)*								
Initial BW, kg	21.7	21.3	21.1	22.3	0.75	0.73	0.83	0.24
Final BW, kg	39.9	39.3	39.0	39.8	1.45	0.78	0.88	0.69
ADG, kg/day	0.87	0.86	0.85	0.83	0.05	0.87	0.95	0.74
ADFI, kg/day	1.67	1.63	1.59	1.71	0.08	0.73	0.76	0.30
G:F ratio	0.52	0.53	0.54	0.49	0.02	0.86	0.58	0.03
*Phase 2 (Days 22 to 29)*								
Initial BW, kg	39.9	39.3	39.0	39.8	1.45	0.78	0.88	0.69
Final BW, kg	47.7	43.5	42.0	42.2	1.66	0.09	0.52	0.92
ADG, kg/day	1.11	0.60	0.43	0.34	0.10	0.0020	0.24	0.57
ADFI, kg/day	2.40	1.68	1.56	1.41	0.12	0.0002	0.50	0.41
G:F ratio	0.47	0.34	0.23	0.19	0.06	0.13	0.24	0.66

^1^ G = Group of pigs. Phase-1 values were obtained before diquat administration, and Phase-2 values were obtained after diquat administration. ^2^ Diquat was administrated on day 22 of the experiment. ^3^ *p*-values were obtained from the ANOVA test with three preplanned orthogonal contrasts.

**Table 4:** Effects of dietary supplementation of DL-Met or MHA-Ca on the serum and liver malondialdehyde (MDA) contents (µM) in the growing pigs ^1^.

		Diquat Challenged ^2^				Contrast *p*-Value ^3^		
Item	G 1	G 2	G 3	G 4	SEM	G 2 vs. G 1	G 3 vs. G 2	G 4 vs. G 3
*Serum*								
Day 1	5.19	5.47	5.03	4.78	0.54	0.71	0.57	0.74
Day 22	4.96	4.92	5.36	4.68	0.58	0.96	0.60	0.41
Day 29	5.66	8.34	8.80	7.25	0.73	0.01	0.66	0.14
*Liver*								
Day 29	2.47	2.15	2.37	2.48	0.17	0.19	0.36	0.66

^1^ G = Group of pigs. The day 1 and day 22 values were obtained before diquat administration, and the day 29 values were obtained after diquat administration. ^2^ Diquat was administrated on day 22 of the experiment. ^3^ *P*-values were obtained from the ANOVA test with three preplanned orthogonal contrasts.

**Table 5:** Effects of dietary supplementation of DL-Met or MHA-Ca on the serum total antioxidant capacity (TAC, mM TE) of growing pigs ^1^.

		Diquat Challenged ^2^				Contrast *p*-Value ^3^		
Item	G 1	G 2	G 3	G 4	SEM	G 2 vs. G 1	G 3 vs. G 2	G 4 vs. G 3
Day 1	5.46	5.33	5.50	5.50	0.17	0.60	0.48	0.98
Day 22	9.04	9.39	9.21	9.47	0.16	0.11	0.41	0.24
Day 29	12.5	12.3	12.6	12.4	0.11	0.14	0.05	0.15

^1^ TE = Trolox equivalent. G = group of pigs. The day 1 and day 22 values were obtained before diquat administration, and the day 29 values were obtained after diquat administration. ^2^ Diquat was administrated on day 22 of the experiment. ^3^ *p*-values were obtained from the ANOVA test with three preplanned orthogonal contrasts.

**Table 6:** Effects of dietary supplementation of DL-Met or MHA-Ca on the serum glutathione (GSH) content (µM) in growing pigs ^1^.

		Diquat Challenged ^2^				Contrast *p*-Value ^3^		
Item	G 1	G 2	G 3	G 4	SEM	G 2 vs. G 1	G 3 vs. G 2	G 4 vs. G 3
Day 1	25.9	21.3	19.7	16.0	2.22	0.61	0.84	0.46
Day 22	22.8	28.1	40.5	31.2	2.87	0.49	0.11	0.25
Day 29 ^4^	n.a.	n.a.	n.a.	n.a.	n.a.	n.a.	n.a.	n.a.

^1^ G = group of pigs. The day 1 and day 22 values were obtained before diquat administration, and the day 29 values were obtained after diquat administration. ^2^ Diquat was administrated on day 22 of the experiment. ^3^ *p*-values were obtained from the ANOVA test with three preplanned orthogonal contrasts. ^4^ The day 29 values (n.a. = not analyzed) were not available because a few serum samples had been run out.

**Table 7:** Effects of dietary supplementation of DL-Met or MHA-Ca on the serum and liver glutathione peroxidase (GPX) activity (nmol/min/mL) in growing pigs ^1^.

		Diquat Challenged ^2^				Contrast *p*-Value ^3^		
Item	G 1	G 2	G 3	G 4	SEM	G 2 vs. G 1	G 3 vs. G 2	G 4 vs. G 3
*Serum*								
Day 1	82.6	90.3	100.4	98.7	6.75	0.45	0.30	0.85
Day 22	102.6	106.8	106.6	105.9	8.26	0.72	0.99	0.95
Day 29	119.3	106.8	135.0	123.4	4.53	0.06	<0.0001	0.08
*Liver*								
Day 29	19.5	15.9	20.8	20.8	1.46	0.10	0.02	0.99

^1^ G = group of pigs. The day 1 and day 22 values were obtained before diquat administration, and the day 29 values were obtained after diquat administration. ^2^ Diquat was administrated on day 22 of the experiment. ^3^ *p*-values were obtained from the ANOVA test with three preplanned orthogonal contrasts.

**Table 8:** Effects of dietary supplementation of DL-Met or MHA-Ca on the serum and liver superoxide dismutase (SOD) activity (U/mL) in growing pigs ^1^.

		Diquat Challenged ^2^				Contrast *p*-Value ^3^		
Item	G 1	G 2	G 3	G 4	SEM	G 2 vs. G 1	G 3 vs. G 2	G 4 vs. G 3
*Serum*								
Day 1	5.35	5.49	4.90	4.68	0.53	0.85	0.40	0.76
Day 22	4.27	5.29	5.46	4.58	0.74	0.35	0.87	0.39
Day 29	5.65	5.49	5.22	5.18	0.39	0.84	0.74	0.96
*Liver*								
Day 29	0.73	0.51	0.71	0.75	0.05	0.01	0.08	0.79

^1^ G = group of pigs. The day 1 and day 22 values were obtained before diquat administration, and the day 29 values were obtained after diquat administration. ^2^ Diquat was administrated on day 22 of the experiment. ^3^ *p*-values were obtained from the ANOVA test with three preplanned orthogonal contrasts.

**Table 9:** Effects of dietary supplementation of DL-Met or MHA-Ca on the serum and liver catalase (CAT) activity (nmol/min/mL) in growing pigs ^1^.

		Diquat Challenged ^2^				Contrast *p*-Value ^3^		
Item	G 1	G 2	G 3	G 4	SEM	G 2 vs. G 1	G 3 vs. G 2	G 4 vs. G 3
*Serum*								
Day 1	49.4	43.0	44.4	44.2	2.52	0.39	0.57	0.94
Day 22	67.1	62.6	54.1	51.6	2.72	0.25	0.03	0.53
Day 29	67.8	58.1	59.3	59.2	6.10	0.27	0.90	0.99
*Liver*								
Day 29	26.6	23.4	21.9	22.3	2.53	0.37	0.67	0.91

^1^ G = group of pigs. The day 1 and day 22 values were obtained before diquat administration, and the day 29 values were obtained after diquat administration. ^2^ Diquat was administrated on day 22 of the experiment. ^3^ *p*-values were obtained from the ANOVA test with three preplanned orthogonal contrasts.

## Data Availability

The original contributions presented in this study are included in the article/Supplementary Materials. Further inquiries can be directed to the corresponding author.

## Supplementary Materials

The following supporting information can be downloaded at: https://www.mdpi.com/article/10.3390/ani14233397/s1. Table S1. The analyzed nutrient and energy composition of the three experimental diets, as-fed basis.
