# Supplementary material for: Effects of Dietary Supplementation of DL-Methionine or DL-Methionine Hydroxyl Analogue (MHA-Ca) on Growth Performance and Blood and Liver Redox Status in Growing Pigs
Source: Animals (Basel). 2024 Nov 25;14(23):3397. doi: 10.3390/ani14233397 (PMC11640123; doi:10.3390/ani14233397)
Supplement: Supplementary file 1 [file animals-14-03397-s001.zip › animals-3316094-supplementary.pdf]

Supplemental Table S1. The analyzed nutrient and energy composition of the three experimental diets[1], as-fed basis

Nutrient item[2]	Diet 1	Diet 2	Diet 3
Dry matter	88.1	87.9	87.9
Gross energy, kcal/kg	3,840	3,830	3,829
Crude protein	17.6	17.4	16.9
Crude fiber	1.95	1.68	2.07
Neutral detergent fiber	9.30	10.26	9.57
Acid detergent fiber	2.67	2.92	2.58
Ether extract	2.61	2.75	2.81
Ash	4.52	4.73	4.25
Lysine	1.12	1.10	1.20
Lysine, Supplemental	0.35	0.38	0.37
Methionine + cysteine	0.73	0.85	0.56
Methionine, Supplemental	0.13	0.30	n.d.
MHA-Ca, Supplemental	n.d.	n.d.	0.54
Threonine	0.75	0.74	0.76
Threonine, Supplemental	0.09	0.13	0.15
Tryptophan	0.23	0.22	0.23
Arginine	1.08	1.05	1.04
Isoleucine	0.70	0.69	0.65
Leucine	1.51	1.50	1.40
Valine	0.80	0.79	0.76
Histidine	0.45	0.44	0.43
Phenylalanine	0.84	0.84	0.79
Glycine	0.70	0.69	0.67
Serine	0.84	0.82	0.79
Proline	1.04	1.04	0.99
Alanine	0.92	0.91	0.86
Aspartic acid	1.64	1.61	1.55
Glutamic acid	3.05	3.01	2.86
Ammonia (NH_3)	0.37	0.36	0.34

[1]The unit for all these nutrient items is percentage (%), except for the gross energy whose unit is kcal/kg.

[2]While the energy and proximate analyses were conducted at the Essig Animal Nutrition Laboratory, Mississippi State University (Starkville, MS, USA), the amino acid contents were analyzed with the methods based on the near-infrared (NIR) spectroscopy at the analytical laboratory of Evonik Nutrition & Care GmbH (Hanau-Wolfgang, Germany). n.d. = not detected.
